# Effectiveness of a drug dosing service provided by community pharmacists in polymedicated elderly patients with renal impairment — a comparative study

**DOI:** 10.1186/1471-2296-14-96

**Published:** 2013-07-13

**Authors:** M Angeles Via-Sosa, Natali Lopes, Marian March

**Affiliations:** 1 Pharmacy Practice. Unidad de Prácticas Tuteladas. Faculty of Pharmacy, University of Barcelone, Spain; 2 Master in Pharmaceutical Care. Faculty of Pharmacy, University of Barcelone, Spain

**Keywords:** Drug dosing service, Community pharmacist, Renal impairment, Polymedication, Elderly patients

## Abstract

**Background:**

Drug dosing errors are common in renal-impaired patients. Appropriate dosing adjustment and drug selection is important to ensure patients’ safety and to avoid adverse drug effects and poor outcomes. There are few studies on this issue in community pharmacies. The aims of this study were, firstly, to determine the prevalence of dosing inadequacy as a consequence of renal impairment in patients over 65 taking 3 or more drug products who were being attended in community pharmacies and, secondly, to evaluate the effectiveness of the community pharmacist’s intervention in improving dosing inadequacy in these patients when compared with usual care.

**Methods:**

The study was carried out in 40 Spanish community pharmacies. The study had two phases: the first, with an observational, multicentre, cross sectional design, served to determine the dosing inadequacy, the drug-related problems per patient and to obtain the control group. The second phase, with a controlled study with historical control group, was the intervention phase. When dosing adjustments were needed, the pharmacists made recommendations to the physicians. A comparison was made between the control and the intervention group regarding the prevalence of drug dosing inadequacy and the mean number of drug-related problems per patient.

**Results:**

The mean of the prevalence of drug dosing inadequacy was 17.5% [95% CI 14.6-21.5] in phase 1 and 15.5% [95% CI 14.5-16.6] in phase 2. The mean number of drug-related problems per patient was 0.7 [95% CI 0.5-0.8] in phase 1 and 0.50 [95% CI 0.4-0.6] in phase 2. The difference in the prevalence of dosing inadequacy between the control and intervention group before the pharmacists’ intervention was 0.73% [95% CI (−6.0) - 7.5] and after the pharmacists’ intervention it was 13.5% [95% CI 8.0 - 19.5] (p < 0.001) while the difference in the mean of drug-related problems per patient before the pharmacists’ intervention was 0.05 [95% CI( -0.2) - 0.3] and following the intervention it was 0.5 [95% CI 0.3 - 0.7] (p < 0.001).

**Conclusion:**

A drug dosing adjustment service for elderly patients with renal impairment in community pharmacies can increase the proportion of adequate drug dosing, and improve the drug-related problems per patient. Collaborative practice with physicians can improve these results.

## Background

Chronic Kidney Disease (CKD) is an important health problem with high incidence and prevalence and a close association with cardiovascular diseases and diabetes [1,2]. In the United States, the estimated prevalence for chronic renal impairment in adults is 13% [3] and in the Spanish population it is 6.8% (ages ≥ 20) and 21.4% (ages > 64) [4]. In patients with hypertension and diabetes, the prevalence of CKD can reach up to 35-40% [5] and, in the elderly, it rises exponentially [6].

Inadequate dosing adjustment in renally excreted drugs is one of the main causes of iatrogenesis [7]. Unsuitable dosing or frequency of administration is considered be responsible for about 70-75% errors in these drugs [8].

A drug-related problem (DRP) is defined as *an event or circumstance involving drug therapy that actually or potentially interferes with desired health outcomes*[9].

About one third of hospital admissions as a consequence of a DRP have been registered in patients over 60 years [10] and half of these are due to adverse drug events (ADEs) [11].

DRPs are the fourth cause of elderly mortality in the USA and have a negative impact on mortality, morbidity, functionality, and resource availability and use [11].

Pharmacist care tries to ensure the effective and safe use of drugs for patients to enhance their quality of life. Safety is a key element in avoiding side effects through early detection of DRPs.

Studies conducted in hospital settings and residential homes in which the pharmacist tried to prevent and treat DRPs in elderly, renally-impaired patients produced positive results in terms of patients’ health [12-15]. Recently, other studies [16,17] showed a reduction in medication errors and in inappropriately high doses of renally excreted medications in patients with CKD in ambulatory or primary care settings. However, according to a recent systematic review [18], no studies have been conducted in community pharmacies.

The aims of this study were to determine the prevalence of dosing inadequacy as a consequence of renal impairment in patients over 65 that were taking 3 or more drug products and who were being attended in community pharmacies, and to evaluate the effectiveness of the community pharmacist intervention in addressing the problem of dosing inadequacy as a consequence of renal impairment in patients over 65 years that were taking 3 or more drugs when compared with usual care.

## Methods

### Design

**Figure 1:**
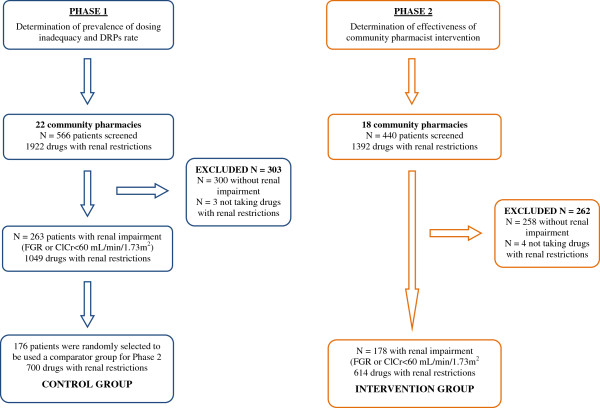
General schema of study.

This study consists of two phases (Figure 1):

Phase 1: To determine the prevalence of dosing inadequacy, an observational, multicentre, cross-sectional study was carried out.

Phase 2: To evaluate the effectiveness of the community pharmacist intervention, a non-randomised controlled study with historical control group was carried out. The Ethics Committee at Germans Trias i Pujol Hospital revised and approved the study (EO-12-038).

### Setting and participants

The study was carried out by 40 volunteer pharmacists in 40 community pharmacies, accredited by the University of Barcelona for training senior pharmacy students, from various areas of Barcelona (Spain): Barcelonès nord, Maresme, Vallès occidental, Vallès oriental, Berguedà and Osona. The participant pharmacists received two sessions (8 hours) of training prior to the study in which they were trained to detect a patient with renal impairment and make a dosage adjustment if needed.

Patients over 65 years old that presented to one of the participant community pharmacies with 3 or more prescriptions were invited to participate in the trial by the pharmacists (Phase 1: Oct 2010-Jan 2011; Phase 2: Feb 2011-May 2011). The inclusion criteria were: people over 65 years old; taking 3 or more drug products; having a Body Mass Index (BMI) between 19 and 35 Kg/m^2^; not being vegetarian or on a high protein diet; not having had a limb amputated, paralysis or muscular diseases; not taking creatinine-affecting drugs. Written informed consent was obtained for all participants.

Participants were screened by pharmacists (a minimum of 25 participants screened per pharmacist) for renal impairment by measuring the glomerular filtration rate (GFR) and creatinine clearance (CrCl). Serum creatinine values were used to calculate the CrCl (Cockcroft-Gault method) and the glomerular filtration rate (Modification of Diet in Renal Disease MDRD-4 method) with the help of an online renal calculator [19]. Patients with GFR levels lower than 60 ml/min/1.73 m^2^ and/or a CrCl lower than 60 ml/min were considered as having a renal impairment and were included in the study (Figure 1).

### Variables

Data on variables were collected by the pharmacist in an interview with the patient at the community pharmacy.

*Sociodemographic variables*: age and gender; *anthropometric variables*: Height (m), measured through calibrated height scales; weight (Kg): measured through calibrated weight scales; Body Mass Index (BMI): in kg/m^2^; Body surface area (m^2^) calculated through Mosteller’s method [19]. *Clinical Variables*: Blood Pressure (BP) (mmHg), measured through calibrated and validated instruments; Serum creatinine (SCr) in mg/dL through Reflotron® or blood test conducted in the previous 3 months at the Primary Care Health Centre; comorbidity (hypertension, diabetes, dyslipidemia, hyper/hypo thyroidism, cardiovascular disease (CVD) reported by the patient). Pharmacological treatment: anatomical and therapeutic and chemical group (ATC), active ingredient, doses, dosage, and dosing interval.

### Intervention

Certain drug databases, such as CIMA [20] (Medicines Online Information Centre) the Spanish official drugs Information, Martindale [21] and American Hospital Formulary Drug System Information [22] (AHFS), were consulted to study the dosing inadequacy of the active ingredients contained in the drug products that the patients were taking. When an active ingredient requiring dose adjustment (Cause of DRP Code C3.2, C3.4 of Classification of Pharmaceutical Care Network Europe Foundation PCNE) [9] was detected or when it was contra-indicated (Code C1.1 of PCNE) [9], we considered the patient as having a potential DRP (PCNE Code P2.1, P2.3) [9].

The prevalence of dosing inadequacy (DI) was calculated through the mathematical expression [12]: DI = (N° inadequately adjusted and/ or contra-indicated drugs / N° total adjustable drugs)*100

Pharmacists used a questionnaire prepared by the research team to write a brief report to the general practitioner (GP) detailing the problems that had been detected and suggesting changes in pharmacological treatment (change drug, decrease quantity of dosage, lengthen time interval between doses). In this written report there was a space that allowed the GPs to provide the pharmacists with a written answer. These written reports were delivered to the GPs by the pharmacists (face-to-face or using the GP’s mailboxes). After 7–14 days the pharmacists went to the Primary Care Health Centre (PCHC) to collect any replies from the GPs. The reports included the pharmacists’ telephone numbers to allow the GPs to contact them.

### Sample size calculation

In Phase 1 the objective was to determine the prevalence of dosing inadequacy in the elderly polymedicated population with renal impairment. Assuming that the prevalence of dosing inadequacy in the elderly is 20%, as has been previously reported [12], and to obtain a precision of 3% with a 95% confidence interval, the sample size required was 683 drug products with restrictions in case of renal impairment.

In the second phase, the aim was to evaluate the effectiveness of the community pharmacist intervention in addressing the problem of dosing inadequacy when compared with usual care. Taking into account expected dosing inadequacy values of 20% in the control group and 10% in the intervention group and to detect differences between groups in the levels of dosing inadequacy with a power of 95% and a 95% confidence interval, a minimum of 328 drug products with restrictions in case of renal impairment per group was required.

The number of drug products needed to assess the prevalence of requirements for dosing adjustments was higher than the number of drugs needed to evaluate the effectiveness of the pharmacist intervention. Consequently, 176 patients from the 263 that had been included in Phase 1 formed a randomly chosen subsample to be used as historical control group in Phase 2 (Figure 1).

### Statistical analysis

Data analysis was performed using the Statistical Package for the Social Sciences version 19 (SPSS). Quantitative variables were expressed as means. Other variables were expressed as relative and absolute frequencies. The comparison between variables was conducted using the Chi-Squared test and Student’s t-test for qualitative and quantitative variables, respectively. ANOVA was also used to compare qualitative variables for multiple layer quantitative analyses. P values less than 0.05 were considered statistically significant. Population parameter estimates were carried out at a Confidence Interval (CI) of 95%.

## Results

### Phase 1: cross sectional descriptive study

In the first phase, 22 community pharmacies and 566 patients participated. Glomerular filtration analysis showed 266 patients with some degree of renal impairment (CrCl < 60 ml/min and/or GFR < 60 ml/min/1.73 m^2^) in the screening performed by pharmacists. Three patients were excluded because they were not taking any drug with restrictions in the case of renal impairment (Figure 1). Table 1 shows the patients’ baseline characteristics by gender.

**Table 1:** **Patient demographic and clinical characteristics at baseline according to gender** (**Phase 1**)

**Mean ± SD**	**Women(173)**	**Men(90)**	**Overall(263)**
Age (years)	83.1 ± 7.2	82.6 ± 6.9	82.9 ± 7.1
Weight (kg)	62.9 ± 11.6	73.9 ± 10.1	69.8 ± 12.2
Height (m)	1.5 ± 0.1	1.6 ± 0.1	1.6 ± 0.1
BMI (Kg/m^2^)	26.4 ± 4.1	27.0 ± 3.2	26.6 ± 3.9
SBP (mm Hg)	130.6 ± 17.5	130.9 ± 17.7	130.7 ± 17.5
DBP (mm Hg)	70.8 ± 11.1	71.7 ± 11.3	71.1 ± 11.2
Creatinine (mg/dl)	1.1 ± 0.9	1.30 ± 0.4	1.2 ± 0.8
Body surface (m^2^)	1.6 ± 0.2	1.8 ± 0.1	1.7 ± 0.2
GFR (ml/min/1.73 m^2^)	61.0 ± 16.9	59.2 ± 16.1	60.4 ± 16.7
CrCl (ml/min)	43.0 ± 11.7	47.5 ± 12.9	45.2 ± 12.2
CrCl-BS (ml/min/1.73 m^2^)	46.7 ± 11.6	44.5 ± 10.9	45.0 ± 11.4
Total number drugs patient	7.3 ± 3.0	7.2 ± 2.9	7.3 ± 2.9
Drugs with restrictions per patient	4.0 ± 1.9	4.0 ± 2.9	4.0 ± 2.0
Number of DRP per patient	0.7 ± 1.1	0.6 ± 1.1	0.7 ± 1.1
Prevalence of DI (%)	18.3 ± 28.9	15.8 ± 28.4	17.5 ± 28.2
Population with DRP %(n)	35.6(32)	38.2(66)	37.3(98)
Comorbidity %(n)			
Hypertension	71.7%(124)	67.8%(61)	70.3%(185)
T2DM	19.7%(34)	24.4%(22)	21.3%(56)
Dyslipidemia	37.0%(64)	36.7%(33)	36.9%(97)
Hyper/Hypo Thy	8.1(14)	1.1%(1)	5.7%(15)
CVD	26.6(46)	31.1%(28)	28.1%(74)

Abbreviations: *BMI* Body Mass Index, *SBP* Systolic Blood Pressure, *DBP* Diastolic Blood Pressure, *T2DM* Type 2 Diabetes Mellitus, *CVD* Cardiovascular Disease, *THY* Thyroidsm, *GFR* Rate of Glomerular filtration, *CrCl* Creatinine Clearance, *CrCl*-*BS* Creatinine Clearance adjusted to body surface, *RI* renal impairment, *DRP* Drug-related problem, *DI* Dosing inadequacy, Drugs with restrictions (dose adjustment needed in case of renal impairment (RI)) per patient [20-22], *SD* Standard deviation.

The 263 patients included were taking a total of 1,922 drug products of which 1,049 [54.6% 95% CI 52.3-56.7] may have dosing restrictions or be contraindicated depending on patient’s renal functioning. The mean of the prevalence of dosing inadequacy in this group was 17.5% [95% CI 14.6 - 21.4] and the mean of DRPs per patient was 0.7 [95% CI 0.5-0.8] (Table 1).

**Figure 2:**
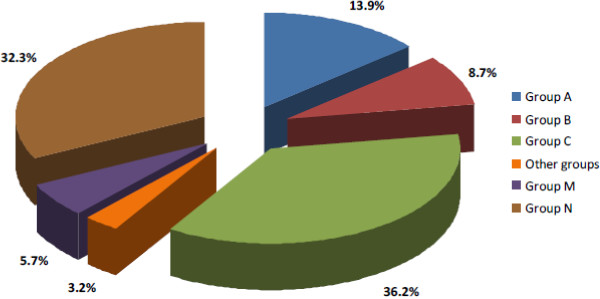
Breakdown of percentage Anatomical Groups (ATC).

The active ingredients contained in these 1,049 drug products that may have needed dose adjustments or were contra-indicated in case of renal impairment were distributed among eleven anatomical groups (Figure 2). The anatomical group that presented most drugs was group C which corresponds to the Cardiovascular System. Four out of every ten drugs used by patients belonged to this group. Most of the drugs work on the renin-angiotensin system (C09) (ACE inhibitors, Angiotensin II antagonists) and were followed by diuretics (C03) (thiazides) and HMG-CoA Reductase Inhibitors (statins) C10. The N group refers to the Nervous System and also presented a high prescription percentage; three of every ten drugs belonged to this group. The analgesics (N02), psycholeptics (N05) and psychoanaleptics (N06) subgroups were the most frequent. In the A group (Alimentary tract and metabolism) it is notable that the most prescribed drugs were those used to treat diabetes (A10).

### Phase 2: intervention study

In the second phase, 18 pharmacies and 440 patients were included. The intervention group consisted of 178 of these patients who showed some degree of renal impairment. Four of these patients did not take any drugs suitable for dosing adjustment in the case of renal impairment and were excluded from the study. Finally, the intervention was carried out in 174 patients (Figure 1). Table 2 describes the general characteristics according to gender.

**Table 2:** **Patient demographic and clinical characteristics at baseline according to gender** (**Phase 2**)

**Mean ± SD**	**Women (103)**	**Men (71)**	**Overall (174)**
Age (years)	81. ± 7.5	80.5 ± 7.0	80.8 ± 7.3
Weight (kg)	61.9 ± 10.7	73.9 ± 10.1	66.8 ± 12.0
Height (m)	1.5 ± 0.1	1.7 ± 0.1	1.6 ± 0.1
BMI (Kg/m^2^)	26.0 ± 4.2	26.6 ± 3.1	26.3 ± 3.8
SBP (mm Hg)	136.3 ± 1932	132.4 ± 17.8	134.7 ± 18.7
DBP (mm Hg)	75.8 ± 12.0	73.9 ± 12.6	75.0 ± 12.6
Creatinine (mg/dl)	1.0 ± 0.3	1.5 ± 1.1	1.2 ± 0.8
Body surface (m^2^)	1.6 ± 0.2	1.8 ± 0.2	1.7 ± 0.2
GFR (ml/min/1.73 m^2^)	61.3 ± 14.5	59.8 ± 20.0	60.7 ± 17.0
CrCl (ml/min)	45.2 ± 11.2	46.9 ± 15.2	45.9 ± 12.3
CrCl-BS (ml/min/1.73 m^2^)	48.3 ± 11.5	45.2 ± 13.9	47.1 ± 12.6
Total number of drugs per patient	6.3 ± 3.3	6.2 ± 3.0	6.3 ± 3.1
Drugs wit restriction in RI per patient	3.5 ± 2.1	3.5 ± 2.0	3.5 ± 2.1
Number of DRP per patient	0.4 ± 0.9	0.6 ± 1.0	0.5 ± 0.9
Prevalence of DI (%)	18.9 ± 30.7	12.4 ± 24.2	15.5 ± 28.6
Population with DRP %(n)	35.0(36)	19.7(14)	28.7(50)
Comorbidity %(n)			
Hypertension	709.%(73)	67.6%(48)	69.5%(121)
T2Dm	13.6%(14)	8.5%(6)	11.5%(20)
Dyslipidemia	42.%(44)	29.6%(21)	37.4%(65)
Hyper/Hypo/Thy	9.7%(10)	1.4%(1)	6.3%(11)
CVD	19.4%(20)	29.6%(21)	23.6%(41)

Abbreviations: *BMI* Body Mass Index, *SBP* Systolic Blood Pressure, *DBP* Diastolic Blood Pressure; *T2DM* Type 2 Diabetes Mellitus, *CVD* Cardiovascular Disease, *THY* Thyroidism, *GFR* Rate of Glomerular filtration, *CrCl* Creatinine Clearance, *CrCl*-*BS* Creatinine Clearance adjusted to body surface, *RI* renal impairment, *DRP* Drug-related problem, *DI* Dosing inadequacy, Drugs with restrictions (dose adjustment needed in case of renal impairment (RI)) per patient [20-22], *SD* Standard deviation.

These 174 patients were taking a total of 1,092 drug products of which 614 (56.2% [95% CI 53.3-59.8]) may have needed dose adjustments or were contra-indicated according to the patient’s renal functioning.

The mean of the prevalence of dosing inadequacy in this group was 15.5% [95% CI 14.5-16.6] and the mean of the DRPs per patient was 0.50 [95% CI 0.4-0.6] (Table 2).

The distribution of the 614 drugs that show use restrictions and/or dosing adjustment in the case of renal impairment was similar to that in phase one of the study.

Among the active ingredients which caused DRPs, it should be mentioned that the C09 sub-group containing Enalapril and Olmesartan were responsible for 26.4% of DRPs detected and treated. DRPs detected and treated with respect to acetyl salicylic acid (group B01) at 8% and Lormetazepam and Triazolam (group N05) at 9.1% should also be noted. Metformin and Repaglinide (group A10) at 3.4%, Ranitidine (group A02) at 4.6%, Simvastatin (group C10) at 5.7%, Allopurinol (group M04) at 3.4% and Alendronic Acid (group M05) at 2.3% may also be highlighted.

**Figure 3:**
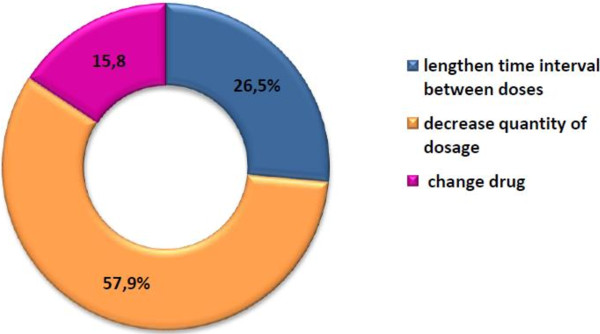
Type of recommendations made to GPs in the intervention group in phase 2.

In the intervention group, pharmacists carried out 167 interventions in 139 patients (1.2 interventions/patient). In 35 patients (25.8%) no intervention was carried out because they did not present DRPs or because they were not considered as suitable for inclusion in a monitoring programme for patients with renal impairment. Some 34 patients (24.5%) were referred to physicians and they carried out 38 dose adjustments. Figure 3 shows the type of recommendations made to GP’s A total of 65.7% of the interventions had no response from the physicians while 31.4% of the interventions were accepted and the DRPs were resolved. Pharmacists included 129 patients with renal impairment in a monitoring programme for patients with renal impairment to examine three issues: 1) placing patients under observation before making dosing adjustments when there was a recommendation in the databases consulted; 2) observing the prescription of new drugs that may require dosing adjustments or surveillance and 3) monitoring of any changes in renal impairment where dosing adjustment is needed.

In the intervention group, the number of DRPs per patient fell from 0.5 at the beginning to 0.2 after the pharmacists’ intervention; showing a difference in the DRPs of 0.3 per patient [95% CI 0.2-0.5] (p < 0.001). Dosing inadequacy also dropped from 15.5% to 5.2%; a 10.4% difference [95% CI 6.5-14.3] ( p < 0.001), and the percentage of patients with DRPs decreased from 28.7% [95% CI 22.0-35.4] at commencement (one out of every three patients) to 11.5% [95% CI 6.8-16.2] (one out of every 10 patients) (p < 0.001).

Finally, we compared the results between the control and intervention groups. Table 3 shows the homogeneity between the control group and the intervention group, allowing comparison between variables that measure the results of the pharmacist intervention. Some statistically significant differences were noted; in age (patients in the control group were 3 years older than patients in the intervention group), in diastolic blood pressure (it was higher in the intervention group), in the number of diabetics (higher in the control group) and in the number of total drug products per patient (higher in the control group). Table 4 shows the results when comparing the prevalence of dosing inadequacy and DRPs per patient between the control and intervention groups adjusted by age, diastolic blood pressure, creatinine, number of total drugs and comorbidity: T2DM. Statistically significant differences were noted (p < 0.001) between the control and intervention groups in the prevalence of dosing inadequacy and in the DRP rate per patient following the pharmacists’ interventions.

**Table 3:** Comparison between general characteristics of control group and intervention group

			**Control group**	**Intervention group**	**P**
Sex		%(n)			0.75^a^
	Women		68.7 (121)	59.2(103)	
	Men		31.3(55)	40.8(71)	
	Overall		100(176)	100.0(174)	
Mean ± SD					
Age (years)			83.3 ± 6.9	80.8 ± 7.3	0.001^b^
Weight (kg)			65.5 ± 12.1	66.8 ± 12.0	0.335^b^
Height (m)			1.6 ± 0.1	1.6 ± 0.1	0.069^b^
BMI (Kg/m^2^)			26.1 ± 4.0	26.3 ± 3.8	0.721^b^
SBP (mm Hg)			130.7 ± 17.3	134.7 ± 18.7	0.060^b^
DBP (mm Hg)			71.5 ± 11.3	75.0 ± 12.3	0.012^b^
Creatinine (mg/dl)			1.1 ± 0.4	1.2 ± 0.8	0.052^b^
Body surface (m^2^)			1.7 ± 0.2	1.7 ± 0.2	0.162^b^
GFR (ml/min/1.73 m^2^)			61.7 ± 16.5	60.7 ± 17.0	0.572^b^
CrCl (ml/min)			45.0 ± 12.1	45. + ±13.0	0.525^b^
CrCl-BS (ml/min/1.73 m^2^)			46.3 ± 11.6	47.1 ± 12.6	0.550^b^
Total number of drugs per patient			7.3 ± 2.9	6.3 ± 3.2	0.003^b^
Drugs with restriction in RI per patient			4.0 ± 2.0	3.5 ± 2.1	0.069^b^
Number of DRP per patient			0.7 ± 1.0	0.5 ± 1.0	0.164^b^
Prevalence of DI (%)			17.5 ± 28.2	15.5 ± 29.1	0.514^b^
Comorbidity %(n)					
Hypertension			72.2(127)	69.5(121)	0.639^a^
T2DM			23.9(42)	11.5(20)	0.003^a^
Dyslipidemia			39.8(70)	37.4(65)	0.662^a^
Hyper/Hypo Thyroidism			5.7(10)	6.3(11)	0.826^a^
CVD			30.7(54)	23.6(41)	0.150^a^
CKD			10.2(18)	8.0(14)	0.579^a^

Abbreviations: *BMI* Body Mass Index, *SBP* Systolic Blood Pressure, *DBP*, Diastolic Blood Pressure, *T2DM* Type 2 Diabetes Mellitus, *CVD* Cardiovascular Disease, *CKD* Chronic Kidney Disease, *GFR* Rate of Glomerular filtration, *CrCl* Creatinine Clearance, *CrCl*-*BS* Creatinine Clearace adjusted to body surface, *RI* renal impairment, *DRP* Drug-related problem, *DI* Dosing inadequacy, Drugs with restrictions (dose adjustment needed in case of renal impairment (RI)) per patient [20-22], *SD* Standard deviation.

^a^χ^2^Test.

^b^ T Student test for independent groups.

**Table 4:** **Comparison between the number of DRPs per patient and the prevalence of dosing inadequacy between control and intervention groups adjusted by age**, **diastolic blood pressure**, **creatinine**, **number of total drugs and comorbidity**: **T2DM**

	**n**	**Mean of difference CI 95%**	**p**
Number of DRPs per patient Pre-INTERVENTION			
Control group	176	Ref	
Intervention group	174	0.05(−0.2-0.3)	0.671
Number of DRPs per patient Post-INTERVENTION			
Control group	176	Ref.	
Intervention group	174	0.50(0.3-0.7)	<0.001
Prevalence of DI Pre-Intervention			
Control group	176	Ref.	
Intervention group	174	0.73(−6.0-7.5)	0.831
Prevalence of DI Post-Intervention I			
Control group	176	Ref.	
Intervention group	174	13.5(8.0-19.5)	<0.001

Abbreviations: *DRP* Drug-related problem (number per patient), *DI* Dosing inadequacy (%), *CI* Confidence Interval, *n* Number of patients, *Ref* Group of reference.

The difference in the prevalence of dosing inadequacy between the control and intervention group before the pharmacists’ intervention was 0.73% [95% CI_0_ (−6.0) -7.5], and after the pharmacists’ intervention it was 13.5% [95% CI 8.0-19.5]. The difference in the DRP rate per patient between the control and intervention group before the pharmacists’ intervention was 0.05 [95% CI (−0.2) - 0.4] and following the intervention it was 0.50 [95% CI 0.3 - 0.7].

## Discussion

The dosing inadequacy caused by renal impairment has been described in the bibliography and shows large oscillations (as also shown in the findings of various authors [14,15,23,24]) which indicates the magnitude of the problem. Our study reveals similar results to other authors on the subject in distinct types of health care institutions. Alvarez Arroyo et al. [12] in a hospital-based study with 185 patients (88 in the control group and 97 in the intervention group), found a frequency of dosing inadequacy of 22.5% in the control group and 18.7% in the intervention group. After the pharmaceutical intervention, the frequency of dosing inadequacy went down by 2.1% and this resulted in lower drug expenditure. Another study [13], this one in a geriatric nursing home, demonstrated dosing inadequacy of 11% in elderly patients with creatinine clearance below 60 ml/min. After the pharmaceutical intervention, the inadequacy percentage decreased. In this case most of the interventions were accepted by the physicians due to the collaboration between both groups of professionals. In another study on the impact of adjustments in hospitalised patients with CKD [14], with a very similar design to this one, the prevalence of inadequacy was about 53%. After the pharmacists’ interventions this dropped to 27.5%. Several patients showed CKD at stage 5, which explains the great need for dosing adequacy in this case.

A controversial point could be the implication of using MDRD-4 formula versus Crockroft-Gault formula for renal dosing adjustments. Several authors [25-30] have studied the dosing implications of using these two formulae for medication therapy adjustments where differences were noted according to the formula used. We, therefore, used both formulae in this study in a simple and practical way (using the online calculator [19]), and from the value of Serum Creatinine we calculated the CrCl and GFR for both formulations. The therapeutic adjustment was applied according to database values (CIMA [20], Martindale [21], AHFS [22]) which were consulted for this study. No significant differences were noted between the separate databases.

One of the limitations of this study was the low response from the physicians to the pharmacists’ recommendations; only one out of three recommendations was followed. But in the cases where the physicians responded almost all the pharmacists’ suggested interventions were accepted. One explanation might be that Spanish pharmacies are not fully integrated into the public health system and physicians tend to see the pharmacist as a professional outside the system. Rubio-Valera et al. [31] examined the most important factors affecting GP and pharmacist collaboration. Those related to economic issues, management and practitioners’ attitudes and perceptions were shown to influence this collaboration. The effectiveness of the pharmacists suggested interventions in dealing with the issue of dosing inadequacy could have been greater with better collaboration between health professionals. This conclusion is supported by other studies [13-15,32].

This underlines shows a real need for physician/pharmacist collaboration, mainly in community healthcare outside hospitals [33]. Collaboration resolved, as seen in this study, many DRPs as well as reducing dosing inadequacy.

The study design with historical control group does not allow us to gather as much scientific evidence as a randomised clinical trial. Despite this, similar designs have been used in other studies [12-14]. To improve outcome reliability, the unanswered physicians’ questionnaires were taken as negative responses to the pharmacists’ recommendations, the DRP was considered unresolved and was then included in the results pool.

This study emphasizes the need to carry out more accurate monitoring of pharmacological treatment in patients over 65 with renal impairment, and the requirement to check renal functioning in the elderly attending community pharmacies.

## Conclusions

The prevalence of dosing inadequacy in polymedicated patients over 65 years with renal impairment attending community pharmacies is not insignificant. The study shows the effectiveness of a community pharmacist intervention in addressing the problem of dosing adequacy of drug treatment in polymedicated elderly patients over 65 years with renal impairment. A drug dosing service for elderly patients with renal impairment in community pharmacies can detect and solve DRPs. Collaborative practice with physicians can improve these results.

## Abbreviations

ADE: Adverse drug event; DRP: Drug related problem; CI: Confidence interval 95%; T2DM: Type 2 diabetes mellitus; CKD: Chronic kidney disease; EPIRCE: Estudio de la Prevalencia de la Insuficiencia Renal Crónica en España (Study of Prevalence of Chronic Renal Disease in Spain); KDOQI: American kidney disease outcome quality initiative; BMI: Body mass index; BP: Blood pressure; SCr: Serum creatinine; CrCl: Clearance; GFR: Glomerular filtration rate; MDRD: Modification in diet renal disease; CVD: Cardiovascular disease; DI: Dosing inadequacy; ATC: Anatomical, therapeutic and chemical classification of drugs.

## Competing interests

No potential conflicts of interest have been reported for this paper.

## Authors’ contributions

MAVS wrote the article, contributed to discussion, researched data and edited the article. NL wrote the article, contributed to discussion and researched data. MM wrote the article, contributed to discussion and reviewed and edited the paper. All authors approved the final draft of the manuscript.

## Pre-publication history

The pre-publication history for this paper can be accessed here:

http://www.biomedcentral.com/1471-2296/14/96/prepub
